# Changes in leg pain after bilateral fasciotomy to treat chronic compartment syndrome: a case series study

**DOI:** 10.1186/1749-799X-8-6

**Published:** 2013-04-05

**Authors:** Jan Roar Orlin, Jarle Øen, John Roger Andersen

**Affiliations:** 1 Section for Orthopedic Surgery, Department of Surgical Sciences, University of Bergen, Bergen, Norway; 2 Department of Anesthesiology, Førde Central Hospital, Førde, N-6807, Norway; 3 Department of Health studies, Sogn and Fjordane University College, Førde, N-6803, Norway; 4 Department of Orthopedics, Førde Central Hospital (FSS), Førde, N-6807, Norway

**Keywords:** Pain, Quality of life, Fasciotomy and Chronic Compartment Syndrome

## Abstract

**Background:**

Intracompartmental pressure (ICP) as the diagnostic gold standard in the management of chronic compartment syndrome (CCS) is debated. We present a diagnostic protocol in which the decision to operate can be based upon clinical findings alone. The aim of this study was to examine whether patients who underwent surgery for CCS based on clinical findings experienced significant long-term pain relief.

**Methods:**

A standardized clinical examination, including skin sensitivity, was performed in patients with bilateral leg pain and/or cramps. Before and after a symptom-provoking step test, ICPs were measured. The primary outcome was self-reported leg pain measured on a visual analogue scale. Secondary outcomes were satisfaction with the treatment result and health-related quality of life (HRQL) measured with the SF-8 questionnaire. Postoperative data were collected after 2 years.

**Results:**

Follow-up was completed for 37 of 40 patients. ICP was increased in 80.5% of the compartments examined before surgery, but did not correlate with the degree of leg pain. The remaining compartments were diagnosed as CCS based on clinical findings, despite ICPs below the threshold. Leg cramps occurred in 32 of 37 (86.5%) patients during physical activity and at night. Leg pain improved from a score of 8.0 ± 1.5 to 2.3 ± 2.1, P < 0.001. Satisfaction with the treatment result was reported by 81.1% of the patients, accompanied by normalized HRQL.

**Conclusions:**

The diagnostic protocol led to a fasciotomy in all compartments of both legs, which was associated with substantial and sustained relief of leg pain, improved HRQL, and patient satisfaction.

## Introduction

Chronic compartment syndrome (also known as chronic exertional compartment syndrome) is a disorder that is most often encountered and described in soldiers and athletes [1-5], but it has also been reported in nonathletes [6]. CCS is characterized by leg pain that is serious enough to prohibit running and sometimes even walking. The pathophysiology of CCS is unknown, but reduced microcirculation capacity and increased ICP has been documented [2,7]. Small observational studies provide limited evidence that as many as 13.9% of leg pain of unknown cause may be related to CCS, while as many as 82% of cases referred for suspected CCS may have elevated ICP [1]. CCS has been documented to occur in the anterior and lateral compartments, and surgical treatment has been directed mainly towards the anterior compartment in the presence of appropriate clinical findings. Recently, CCS was reported in the posterior compartments as well [8]. To establish a diagnosis of CCS, measurement of ICP ≥ 15 mm Hg at rest and ≥ 30 mm Hg 1 minute after exercise has been considered necessary [1,3]. However, a recent review suggested that this diagnostic gold standard for CCS is flawed and recommended that clinicians use protocol-specific upper confidence limits to guide their diagnosis [2]. Thus, clinical practice related to referral for fasciotomy often differs. A diagnosis of CCS that requires fasciotomy may be based on clinical findings only [1], but minimal or comprehensive ICP testing is usually also performed [9,10]. Results after fasciotomy are often good, but for lasting pain relief, decompression of an increasing number of leg compartments may be necessary, paralleling the complete compartmental decompression recommended for acute cases. Poor long-term results may be related to incorrect diagnosis or failure to address multiple compartments in the leg simultaneously [11]. Further exploration of the role of ICP as the diagnostic gold standard and other diagnostic approaches in the management of the CCS appear to be indicated.

In this paper, we present a diagnostic protocol in which the decision to operate can be based upon clinical findings alone. The primary aim of this study was to examine whether patients who underwent surgery for CCS, based upon this diagnostic approach, experienced significant long-term pain relief. Satisfaction with the treatment results and health-related quality of life (HRQL), measured with the SF-8 questionnaire, were secondary outcomes.

## Materials and methods

### Study design and patients

In this case series study, 43 patients with suspected CCS in both legs were examined in a 2-year period (August 2007 to November 2009). Twenty-seven patients with unilateral CCS were excluded because inclusion might have led to reduced external validity. Also excluded were 17 patients who, prior to this study, had undergone fasciotomy of three compartments of both legs, but returned during the study period for fasciotomy of both deep posterior compartments, one at a time. The primary inclusion criterion was a diagnosis of CCS in both legs. Exclusion criteria were the presence of vascular disease, arteriosclerosis, and/or neurological diseases. Three patients were excluded, one because of arteriosclerosis and two due to sciatica. Forty patients fulfilled the inclusion criteria and underwent surgery, but only 37 agreed to participate in the study. The present series was derived from a larger group by eliminating patients who had suspected CCS in only one leg or in whom less than four compartments were affected. The patients were seen in the outpatient clinic and were later operated on by one consultant (JRO). Prior to surgery, all available conservative treatments had proven ineffective over a period of years; i.e., medication, physiotherapy, acupuncture, chiropractic, and cognitive treatment. Data were collected from the medical records. The written patient information, methods and data storage of the study was approved by the National Committee for Medical and Health Research Ethics of Norway (trail number: 20091302).

### Diagnostic protocol

The occurrence of pain and cramps in daytime, produced by exertion such as walking or running and relieved by rest, was noted, as well as nocturnal leg cramps, illustrated by pain drawing. Bilateral leg pain was typically characterized by patients as pressure or congestion. The use of medications and the presence of other diseases prior to surgery were assessed from the patients’ medical records as either present or not present. Physical examination included assessments of the spine and hips, the vascular status of the lower extremities, and blood pressure. The presence of systemic diseases was explored. All patients underwent magnetic resonance imaging (MRI) of the spinal canal. ICPs were measured with a water-based transducer in accordance with the user manual (295-1-730 Rev-C, Stryker Instruments) with patients in the supine position, both before and 1 minute after a step test. Patients were asked to alternate quickly between standing high on their heels and on their forefeet for 3 minutes, as if they were walking while remaining in one spot. Pressures were measured in both legs and in three compartments; the deep posterior compartment was omitted due to the risk of deep bleeding weeks prior to surgery. Measurements of postoperative pressure did not seem to be indicated. In four persons, pain led to the step test being halted within 3 minutes; in two, the test had to be prolonged by 1–3 minutes until symptoms were reproduced. Palpation to determine muscle tenderness and examination of skin sensitivity by cotton swab and pin prick were also repeated. Normal dermatome sensitivity proximal to the knee level and reduced sensitivity in the area supplied by the nervus fibularis profundus and the nervus fibularis superficialis indicated CCS of the anterior and/or lateral compartments, whereas reduced sensitivity in the area of the tibial nerve indicated chronic posterior compartment syndrome. Finally, data from the clinical examination were compared with the pressures that were measured. Palpation of the tarsal tunnel and examination of sensitivity in the areas of the plantar nerves were also performed to identify coexistent tarsal tunnel syndrome.

In summary, a diagnosis of CCS required: (1) the occurrence of leg pain, (2) compartment-specific muscle tenderness, and (3) reduced skin sensitivity. If these findings were not confirmed by increased ICP (≥ 15 mm Hg at rest or ≥ 30 mm Hg 1 minute after an exertional step test), the decision to operate was based on clinical findings alone.

### Surgery and postoperative care

**Figure 1:**
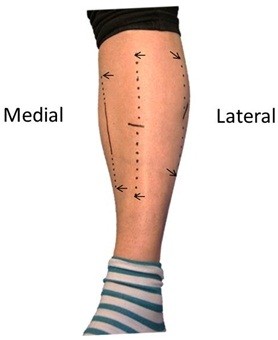
**Photograph of patient’s leg after surgery.** The three fasciotomy incisions used to access all compartments are illustrated. Continuous black lines indicate skin incisions, and dotted lines between arrows indicate the extent of the subcutaneous fascia splitting: approximately 20 cm in the anterior, lateral, and superficial posterior compartments and approximately 10 cm in the deep posterior compartment.

General anesthesia was used except in one case, in which spinal anesthesia was employed. As illustrated in Figure 1, fascia splitting was performed in all four compartments and in one leg at a time through three short skin incisions, an adaptation to modern endoscopic principles. Only the fascia of the anterior compartment was split blindly. The superficial fibular nerve was protected by splitting the fascia under direct vision. The other leg was operated on only after the patient indicated satisfaction with the results of the first operation. The distance between fascial edges was noted. Patients were encouraged to walk from day one and to use elastic stockings for 2 months as protection against phlebitis and deep venous thrombosis (DVT). Follow-ups were performed by someone other than the person who performed the surgery. The mean follow-up time was 2.8 years (range 2–4.3 years). Complications, including hematomas, infections, DVT, and any unintended loss of sensitivity, were registered.

### Patient-reported outcomes

Self-reported leg pain was assessed with a visual analogue scale (VAS) before and after surgery; 0 indicated no pain and 10 the worst possible pain. This method of assessing leg pain has been shown to be valid for patients undergoing surgery for CCS [12]. The patient’s overall satisfaction with the treatment results was assessed by a single-item question: “How satisfied are you, all things considered, with the treatment results after surgery?” The response categories were very satisfied, satisfied, uncertain, and dissatisfied. The validity of assessing patient satisfaction after surgery for painful conditions with this method is considered to be satisfactory [13]. HRQL was measured by the SF-8 questionnaire (QualityMetric), which has been shown to have good validity [14]. This questionnaire assessed the respondent’s recalled health status during the final 4 weeks before surgery and during a 4-week period a minimum of 2 years after surgery. A higher score on the SF-8 scales indicates better health. Gender- and age-adjusted norm scores on the SF-8 scales were derived from a sample of the general population (N = 7,424) [14].

### Statistical analysis

The paired *t*-test was used to study changes in the VAS pain score and SF-8 scores after the operation. The SF-8 scores of the sample were considered to be within the normal range if the means were ± 0.5 SD of those of the general population [15]. The general population SF-8 scores are standardized such that 5 points equals 0.5 SD [14]. Spearman’s rank test was used to study correlations between variables. A P value of ≤ 0.05 was considered statistically significant.

## Results

### Patient characteristics

The participation rate was 92.5% (37 of 40). There were 20 women and 17 men, and the average age was 37.2 ± 16.8 years. CCS was diagnosed in all compartments of both legs in all patients. Data on ICPs were available for 35 patients. Elevated ICPs were found in 80.5% of the compartments examined, and all patients had at least one positive finding of elevated ICP (Table 1). Some compartments had increased pressures at rest, but not after the step test. ICP in the two legs, measured by adding the three ICPs in each leg for a total score, were significantly correlated (Spearman’s rank test = 0.77, P < 0.001). Findings of increased ICP were in all cases in agreement with the clinical criteria. The remaining compartments were diagnosed as CCS, even if the ICP was below the thresholds. Preoperatively, 32 of 37 patients experienced leg pain and cramps during the daytime; 34 of 37 patients had these symptoms at night, causing serious disruption of sleep (Table 2).

**Table 1:** Paired intracompartmental pressures before and after the symptom-provoking step test, expressed as mean values and standard deviations (N = 35)

**Compartments**	**Before step test**	**% with ≥ 15 mm Hg**	**After step test**	**% with ≥ 30 mm Hg**
Left anterior	26.5 ± 15.2	80.6	41.5 ± 22.3	70.0
Left lateral	21.9 ± 7.7	80.6	28.3 ± 16.0	30.0
Left posterior superficial	22.5 ±9.5	74.2	25.2 ± 13.1	30.0
Right anterior	22.8 ± 10.9	81.8	35.3 ± 15.4	58.1
Right lateral	21.0 ± 15.3	63.6	25.3 ± 11.1	32.3
Right posterior superficial	21.7 ± 10.1	74.2	25.6 ± 12.1	30.0

**Table 2:** Prevalence of comorbidities prior to surgery (N = 37 patients)

**Diseases associated with a tendency toward general edema**	**N**	**%**
Hypothyroidism	6	16.2
Diabetes mellitus	3	8.1
Other hormonal changes	5	13.5
Rheumatic disease	4	10.8
Allergy	2	5.4
Heart failure	2	5.4
***Other Conditions***	N	%
Tarsal tunnel syndrome	4	10.8
Carpal tunnel syndrome	8	21.6
Lumbar pain	12	32.4
Growing pain as a child	6	16.2
Insomnia	27	73.0
Trauma by contusion	8	21.6
Deep venous thrombosis	3	8.1

During fasciotomy, the distance between fascial edges was an estimated 1–4 cm: 1.0–1.5 cm in the anterior compartment, 1.5–2.5 cm in the lateral compartment, and 2.0–4.0 cm in the posterior compartment. Postoperatively, increased leg circumference could be observed.

### Patient-reported outcomes

**Figure 2:**
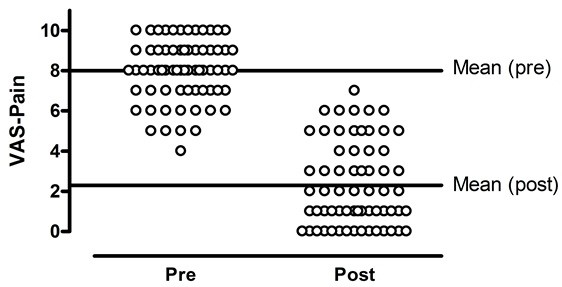
Pre- and postoperative VAS pain scores in both legs (N = 69).

The VAS pain score improved from 8.0 ± 1.5 to 2.3 ± 2.1 (P < 0.001) (Figure 2). No significant correlations were found between the ICP score and the VAS pain score before surgery (Spearman’s rank test = −0.09, P = 0.622). When asked about satisfaction with the overall treatment result, 19 patients were very satisfied, 11 were satisfied, seven were uncertain, and none were dissatisfied. Prior to surgery, the SF-8 scores were much lower than those in the general population, indicating severe impairment. After surgery, the patients’ scores improved significantly and were in the normal range (Table 3). A higher VAS pain score after surgery (Spearman’s rank test = −0.81, P < 0.001) was significantly correlated with lower satisfaction with treatment. No significant correlations were found between treatment satisfaction and ICP before surgery (Spearman’s rank test = 0.09, P = 0.602), or between any of the variables listed in Table 2 (data not shown). The seven patients who were uncertain about satisfaction with the overall treatment result had more pain (as measured by VAS and SF-8) after surgery than the other patients (Mann–Whitney *U*-test, P < 0.05), but their preoperative pressures were not significantly different from the other patients (Mann–Whitney *U*-test, P > 0.05). We also observed that pain relief was achieved by decompression in compartments in which elevated pressure was not found.

**Table 3:** SF-8 scores, preoperative and 2 years after surgery (N = 37)

**SF-8 scores**	**Before surgery**	**After surgery**	**General population**
Physical functioning	32.7 ± 6.4	45.5 ± 7.4*	48.0
Physical role functioning	35.8 ± 9.2	45.7 ± 9.5*	48.3
Bodily pain	31.6 ± 5.8	47.0 ± 8.8*	50.1
General health	35.0 ± 8.7	48.4 ± 8.4*	49.1
Vitality	37.4 ± 8.9	50.8 ± 8.1*	49.4
Social functioning	38.9 ± 9.0	47.7 ± 7.9*	47.9
Emotional role functioning	38.6 ± 9.5	46.9 ± 8.1*	46.5
Mental health	39.8 ± 10.7	50.0 ± 7.0*	47.9

SF-8 scores are presented as mean ± SD for the patients and as means for the general population (N = 7,472).

*P < 0.001.

### Complications

Postoperatively, there was a hematoma in one leg and a DVT in one leg. Prior to surgery, two women, aged 16 and 26 years, had been incapable of even walking. Immediately after surgery, they both increased their physical activity from hardly any to extensive walking, running, dancing, and mountain climbing, resulting in physical overactivity that necessitated months of physiotherapy and medication to treat foot and leg tenoitis and tenoperiostitis.

## Discussion

CCS was diagnosed in all leg compartments of the patients in this series. Leg cramps appeared to be associated with CCS. Satisfaction with the treatment result was reported by 81.1% of the patients. The mean improvement in leg pain as measured by VAS was 5.7 points; this result is statistically significant and represents considerable improvements in quality of daily life. The seven patients who were uncertain about satisfaction with the overall treatment result had more pain after surgery, but their preoperative ICPs were not different from those of the other patients.

To the best of our knowledge, this study was the first to evaluate patient-related outcomes in a general population of patients with CCS and improvements after fasciotomy of all leg compartments. Our findings suggest that decompression of most compartments is indicated more often than we had anticipated prior to the current study; however, clinical findings must be present before the decision is made to perform extensive decompression surgery. That decompression of most or all compartments should be performed more often than indicated by current guidelines is supported by the fact that 17 patients, who were excluded from this study because they had previously undergone fasciotomy of three compartments of both legs, returned to undergo surgery for decompression in the last two compartments.

Although our results appear to be promising, the study has limitations, such as the lack of randomization and the lack of ICP measurements for the deep posterior compartments. Furthermore, muscle tenderness upon palpation is a subjective parameter; however, when muscle tenderness in the lower leg was compared to that above the knee, the difference seemed marked. Our sample may also differ from those presented by others. Thus, further studies should be performed to determine the external validity of our findings.

Our diagnostic approach requires the presence of compartmental tenderness upon palpation combined with decreased sensitivity of one or both rami of the fibular nerve and/or the plantar nerves. If these signs are absent from the outset, an exertional step test may provoke them. Frequently, distinct tenderness was initially present in one anterior leg compartment only, but after the step test, increased muscle pain occurred in all compartments. Often, anticipated referred pain, as depicted in pain drawings, extended towards the knee, hip, and lumbar region; this pain mostly disappeared after both legs had been operated on.

The sensitivity of dermatomes proximal to the knee was compared to that of the innervated areas of the peripheral nerves in the forefoot. Sensitivity changes in these areas would be expected in patients with both a spinal lesion and leg cramps. In our experience, even CCS patients without positive findings on spinal MRI can develop spinal problems later on. In particular, a spinal lesion should be suspected if symptoms occur in a single compartment other than the anterior compartment. One must discriminate between CCS and L5 nerve root impingement as the cause of tender muscles and pain in the anterior compartment. Nonspecific peripheral neuritis must also be eliminated; however, coexisting neuritis could also camouflage symptoms of CCS.

Information about leg cramps associated with CCS is scarce in the literature, but it has been reported before [8]. In the present study, leg cramps were reported by 32 of 37 patients (86.5%); they increased with exertion during the day and caused disturbed sleep at night. After fasciotomy, all patients experienced quick and significant relief from both leg pain and cramps, nocturnal as well as daytime, indicating a correlation between CCS and leg cramps. The fact that only three patients were excluded from this study due to other causes of leg cramps and leg muscle pain indicates that CCS was the predominant cause of these symptoms.

Coexistent tarsal tunnel syndrome, which can cause pain or cramps in plantar muscles similar to that caused by CCS, was recognized by palpation of the tarsal tunnel combined with findings of reduced sensitivity in the areas of the medial and/or lateral plantar nerves; the diagnosis was subsequently confirmed by ultrasound [16]. Concomitant median nerve compression in the arms and other nerve entrapments in the leg are among the additional conditions listed in Table 2. Hormonal disorders were notably common; these disorders have edema as a common denominator, which may thus be a cause of CCS. In previous studies, fasciotomy has had a particularly significant effect on leg pain in diabetics [17].

In the opinion of regional vascular surgeons, because of the low average age of these patients, palpation of arteries in the foot, ankle, and groin, together with inspection of varicose veins, was sufficient to assess the vascular status of the legs. The symptoms of claudicatio intermittens are clearly different from those of CCS.

Measurement of ICP at rest and during exercise has been considered necessary to establish a diagnosis of CCS, but these methods are complex and often yield uncertain results [2]. Moreover, ICP measurements have been performed convincingly by a number of authors who all observed that reduced skin sensitivity resulted from a pressure of ≥ 30 mm Hg [18-20]. Others have suggested that the critical pressure is < 30 mm Hg and that a prolonged period of time from the end of a step test until normalization of pressure is a more important diagnostic factor [20-23].

It is possible that there were errors in the ICP measurements. However, the ICP measurements of Pedowitz et al. [21] were strikingly similar to ours, suggesting that the ICP values are valid. The values in the present study reveal that pressures at rest are more valid than pressures 1 minute after the step test because more patients had elevated pressure at rest than after the step test. The surgical practice of making one large skin incision that provides access to all compartments has been reported [24]. In the present study, small separate skin incisions allowed for decompression of all compartments (Figure 1). In our opinion, the measured distance between fascial edges (1.0–4.0 cm) confirmed the need for volume expansion, which was related to increased leg circumference later on.

## Conclusions

Clinical examinations revealed symptoms of CCS in all four compartments of both legs, while elevated pressures were found in 80.5% of the compartments measured. Statistically, there was no association between leg pain and the degree of ICP. Pain relief was also achieved in decompressed compartments without elevated pressure. The prognosis based on patient-related outcome after fasciotomy on all compartments in both legs was good in 81.1% of the patients. Thus, our data support previous research that suggested clinical examination without pressure measurement may provide an accurate diagnosis of CCS [25]. The difficulties experienced by patients with CCS compared to the general population may provide clues for future muscular pain research. CCS may be underdiagnosed in the general population, and the clinical examination procedure reported here could lead to easier identification of patients with CCS.

## Competing interests

The authors declare that they have no competing interest.

## Authors’ contributions

All the authors participated in the design of the study and in drafting the manuscript. All authors read and approved the final manuscript.

## Authors’ information

Co authors: Jarle Øen and John Roger Andersen.
